# The Effects of Anti-platelets and Micronutrients in the Recovery of COVID-19 Patients: A Review

**DOI:** 10.7759/cureus.42164

**Published:** 2023-07-19

**Authors:** Gurmeet Singh, Nova Fauzi

**Affiliations:** 1 Internal Medicine, Respirology, and Critical Illness, Universitas Indonesia, Jakarta, IDN

**Keywords:** recovery, asprinol, aspirin, micronutrients, covid-19

## Abstract

COVID-19 or coronavirus disease is a pneumonia-like condition caused by the SARS-CoV2 virus. Many mutations of this virus have emerged throughout the two-year period of this pandemic. However, clinical presentations, diagnostic methods, and treatment of COVID-19 remain relatively unchanged. Various substances have been assessed for their functions as COVID-19 immunomodulators. Said substances in this article include aspirin, vitamin C, vitamin D3, zinc, and selenium. Aspirin was found to reduce mortality risk and embolism events. Vitamin C did not seem to improve mechanical ventilation-free days but did improve oxygenation (PaO2/FiO2), peripheral capillary oxygen saturation (SpO2), and body temperature in severe COVID-19 patients. Vitamin D3 was not significantly different compared to placebo in improving mortality in hospitalized patients. However, respiratory tract infection (COVID-19 included) events were lower in individuals given vitamin D3 compared to those who were not. Zinc combined with ascorbic acid caused a quick reduction in symptoms but was not significant compared to zinc alone, ascorbic acid alone, or standard care. Individuals with lower levels of selenium were found to have worse outcomes of COVID-19 compared to those with high levels of selenium. However, further studies, especially clinical trials, are needed. Asprinol is a drug that contains vitamins and minerals plus aspirin which are suggested to help alleviate symptoms and improve outcomes of COVID-19. This review aims to assess the efficacy of asprinol contents in COVID-19 patients.

## Introduction and background

COVID-19 is a pneumonia-like condition caused by the virus SARS-CoV2, and multiple variants have emerged in the two years of this pandemic. The clinical presentations have shown a remarkable degree of consistency, characterized by flu-like symptoms, fever, pneumonia, and acute respiratory distress syndrome. However, with the successful implementation of vaccination efforts, clinical presentations have predominantly shifted toward milder manifestations. In terms of detecting COVID-19 infections, rapid antigen tests and real-time polymerase chain reaction (RT-PCR) swabs continue to be the preferred methods due to their speed and reliability. These tests offer a rapid and accurate means of identification. Treatment options for COVID-19 patients have also not significantly differed. The incidence of mild-moderate cases has exhibited a downward trend, concomitant with the treatment objectives focusing on symptom alleviation through the administration of suitable antiviral medications such as favipiravir or oseltamivir [1]. Immunomodulators are agents that exert an influence on the immune system by modulating immune response and production of antibodies. Extensive study has been conducted to explore the immunomodulatory properties of different substances in the context of COVID-19 [2].

## Review

COVID-19

COVID-19 is caused by SARS-CoV-2, a family member of Coronaviridae. The onset of this disease occurred initially in Wuhan in December 2019, swiftly followed by the subsequent emergence of a second wave in March 2020. During an inopportune period, the WHO officially declared the advent of a pandemic. The transmission of COVID-19 predominantly occurs through respiratory and aerosol droplets. Following its entry into the human body, the virus attaches to specific receptors and gains access to cells through endocytosis or membrane fusion, facilitated by spike proteins. Spike proteins encompass 2 distinct functional subunits, namely S1 and S2. The primary role of the S1 subunit involves binding to the receptors, while the S2 subunit is responsible for facilitating membrane fusion. Angiotensin converter enzyme-2 (ACE-2) is a receptor for this virus, and the receptor is strongly expressed in respiratory epithelial cells. After infiltrating the host cells, the virus releases its genetic material and undergoes replication, thereby generating a new cohort of viral progeny. These newly formed viruses subsequently invade neighboring epithelial and other cellular populations.

During the initial phase, the virus invades the nasal epithelial cells and initiates the replication process. This stage extends over several days, taking advantage of the compromised immune status and the relatively low viral load. The patient does not exhibit any symptoms throughout this duration. Following this, the virus spreads and migrates from the upper respiratory tract through conduction. At this point, the immune system mounts a response, leading to the emergence of symptoms such as fever, malaise, and cough in the patient [3].

In most patients, the virus migrates to the lower respiratory tract and invades type II alveolar epithelial cells expressing ACE-2 receptors. Infected pneumocytes release cytokines and inflammatory markers such as interleukins, tumor necrosis factor (TNF)-a, interferon (IFN)-g, IFN-b, CXCL-10, MCP-1, and MIP-1a, cumulatively. This phenomenon has been termed the cytokine storm, characterized by the chemoattraction of neutrophils, helper T cells, and cytotoxic T cells. Simultaneously, the immune cells provoke inflammation and inflict damage on lung tissues, leading to various complications such as acute respiratory distress syndrome, acute respiratory failure, acute kidney and liver injury, pulmonary embolism, and sepsis [4].

Immunomodulators

Immunomodulators affect the immune response by way of stimulation and suppression, modulating the production of antibodies or rendering tissues unresponsive to antigens. They function at different levels in the immune system, and immune-responsive cells, such as macrophages, lymphocytes, cytotoxic T lymphocytes, and natural killer cells, are inhibited or augmented during the process. Immunomodulators perform either specific or non-specific actions with antigens in the cells. The process becomes specific when stimulations transform into immunoreaction toward one or several antigens, such as therapeutic vaccines or adjuvants. Non-specific immunomodulators, types I-III, work on immune response without regulating the activity of stimulated cells to a particular antigen. Types I and II consist of the normal and suppressed immune system, while type III consists of both. Examples of non-specific immunomodulators include synthetic immunomodulators such as inosine, imiquimod, and products of the immune system itself such as transfer factor, cytokines, IFN, TNF, and granulocyte stimulating factor [5].

Micronutrients in immunomodulation

A balanced, high-quality diet is crucial in achieving optimal immune response and preventing infections. It has been suggested that malnutrition contributes to COVID incidents and complications. There are a few types of malnutrition, namely undernutrition, overweight, obesity, and inadequacy of vitamins or minerals [6].

The viral-host response is dependent on specific nutrients which include vitamins C, D, E, folate, iron, zinc, copper, and selenium. Vitamins C, D, and zinc regulate the fluidity and integrity of the respiratory tract and facilitate gap-junction communication. Selenium regulates the activities of the membrane-bounded antimicrobial peptide and mucosal-related microbiota [7].

Aspirin

Aspirin is a non-steroidal anti-inflammatory drug used to reduce fever and relief mild-to-moderate pain. Furthermore, it lowers the risk of heart attack and stroke by preventing clot formation. Aspirin blocks the production of prostaglandin (PG) and thromboxane (TXA) through irreversible non-activation of cyclo-oxygenase-1 (COX-1) and cyclo-oxygenase-2 (COX-2). Aspirin drug has antiviral effects in vitro by blocking influenza virus propagation through NF-kB inhibition when used at high concentrations and short-term incubation steps [6]. The antiviral properties have been reported in both DNA and RNA viruses, such as cytomegalovirus, rhinovirus, varicella-zoster virus, hepatitis C virus, influenza virus, coxsackie virus, MERS-CoV, and CoV-229E [6,7].

The RECOVERY randomized, controlled, open-label, platform trial assessed the safety and efficacy of aspirin in COVID-19 inpatients between November 1, 2020, and March 21, 2021. The study involved 22,560 patients in 177 hospitals in the United Kingdom, two in Indonesia, and two in Nepal. Furthermore, standard care without and with the addition of 150 mg of aspirin once daily until discharge were compared, with 28-day mortality as the primary outcome. The result showed that 1,299 of 7,541 and 1,222 of 7,351 patients given standard care without and with aspirin were deceased within 28 days. Considering the percentages, approximately 17% of both did not yield a significant difference. However, the patients in the aspirin group had a slightly shorter duration of hospital stay (median: eight days, IQR: 5 to >28 days, vs, nine days, IQR: 5 to >28 days). Among the patients who did not have invasive mechanical ventilation at baseline, there was no significant difference between getting invasive mechanical ventilation and becoming deceased (21% vs. 22%). Standard care with additional aspirin incurred reduced thrombotic events (4.6% vs. 5.3%) but increased bleeding (1.6% vs. 1.0%). Conclusively, in patients hospitalized with COVID-19, aspirin factored in a small increase in being discharged alive and surviving the 28 days [8].

Chow et al. conducted an observational cohort study with a similar design to assess the correlation between early aspirin use and the odds of in-hospital mortality in 112,269 cohorts with moderate COVID-19 across 64 healthcare facilities participating in the National Institute of Health’s National COVID Cohort Collaborative (N3C) in the United States between January 1, 2020, and September 10, 2021. Mortality within 28 days was the primary outcome, while pulmonary embolism and deep vein thrombosis events were secondary. Furthermore, marginal structural Cox and logistic regression models were used to calculate the odds of in-hospital mortality and inverse probability of treatment weighting to reduce confounding bias. The results showed that the 28-day mortality rate was significantly lower in patients who were given aspirin than in those who were not (10.2% vs. 11.8%; OR 0.85; 95% CI, 0.79-0.92; P <0.01). Meanwhile, the incidence of pulmonary embolism was significantly lower in cohorts with aspirin (1% vs. 1.4%; OR 0.71; 95% CI, 0.56-0.90; P= 0.004). Other events of bleeding, including gastrointestinal hemorrhage, cerebral hemorrhage, or blood transfusion were also less frequent [9,10].

In addition, seven studies were systematically reviewed until February 21, 2021, to assess the use of aspirin on the outcome of patients. The main outcome was all mortality, while the secondary consideration was thrombosis and bleeding incidence pooled with DerSimonian-Laird random-effect models. Aspirin use was shown to reduce the risk of mortality (RR 0.56, 95% CI 0.38-0.81, P = 0.002, I2: 68%, P=0.005). Sensitivity analysis of in- and pre-hospital aspirin yielded a significant decrease in heterogeneity (I2: 1%, P = 0.4). Among the seven studies, only one reported a comparison of major bleeding incidence in patients given aspirin [11].

Vitamin C

Vitamin C or ascorbic acid is water-soluble, used as a scavenger for oxygen-free radicals, and co-factor in catecholamine, vasopressin, and cortisol productions. Leukocytes host high levels of vitamin C and facilitate immune responses and functions. Several studies reported that the vitamin was essential in augmenting inflammation effects through pro-inflammatory cytokine production inhibition, immunoregulation, reactive oxygen species neutralization, and host cell protection.

A randomized controlled clinical trial was coordinated at three hospitals in Hubei, China, treating 56 patients with severe COVID-19 in ICUs. Furthermore, the patients were assigned randomly in a 1:1 ratio to high-dose intravenous vitamin C (HDIVC) or placebo. The first group received 12 g of C/50 ml every 12 hours for seven days at 12 ml/hour, while the second had bacteriostatic water for injection within 48 hours of pre-ICU. The primary outcome was invasive mechanical ventilation-free days in 28 days, and the secondary result was 28-day mortality, organ failure, and inflammation progression. The Sequential Organ Failure Assessment score and IL-6 level measurement were used in the secondary outcome. At the end of the seven days, HDIVC failed to decrease the 28-day mortality rate but succeeded in raising PaO2/FiO2 and suppressing IL-6 levels [12].

Another open-label, randomized controlled trial was completed by JamaliMoghadamSiahkali et al. to compare two groups of 30 patients each. Patients with a severe infection in the control group were given lopinavir/ritonavir and hydroxychloroquine, while the case subjects were given HDIVC 6 g. Body temperatures and peripheral capillary oxygen saturation (SpO2) were evaluated on the third day, and the total length of hospital stay was compared. Similarly, the case group had lower mean body temperature, higher SpO2, and longer hospitalization [13].

An assessment of the efficacy of vitamins E and C was also conducted in Iran using a randomized controlled trial on 72 hospitalized, non-severe COVID-19 patients. The infection was confirmed by RT-PCR swab test and CT scan, and the vitamins were administered as adjunctive therapy to the standard treatment regimen. Patients were divided into control and intervention groups of 34 individuals each. They were then randomly allocated the standard treatment consisting of hydroxychloroquine 400 mg on Day 1 and 200 mg every 12 hours or the intervention with oral 1,000 mg of vitamin C and oral 400 IU of vitamin E in addition to the standard regimen. Recording, evaluation, and comparison were applied to clinical response, duration of hospitalization, and mortality rate. However, there was a significant difference between the control and intervention groups. This is because five vs. three patients have treatment failure, while the rest had clinical improvement (P = 0.380). The duration of hospitalization differed between the two groups (8.03 ± 2.83 days vs. 7.95 ± 3.18 days), and there were no patients in either group that died during the study. Therefore, vitamins C and E supplementation to the standard regimen incurred no benefit for non-severe COVID-19 patients [14].

Vitamin D

Vitamin D, known as the sunshine vitamin, is produced in the body after skin exposure to sunlight. It has various benefits for the human body, including proper growth and development of the body, better calcium absorption from the intestines, and skin protection and rejuvenation.

Furthermore, it plays a crucial role in modulating the innate and adaptive immune response. Vitamin D-dependent antimicrobial pathways are induced in response to double-stranded RNA, as produced during SARS-CoV2 replication.

A double-blind, randomized, placebo-controlled trial performed by Murai et al. involved 240 hospitalized moderate-severe COVID-19 patients at two sites in Sao Paolo, Brazil, with 237 patients. The primary outcome was the length of stay, which was described as the time range from randomization to discharge. The subjects were discharged after meeting the following criteria: no supplemental oxygen within the last 48 hours, no fever within the last 72 hours, and oxygen saturation >93% without oxygen supplementation and respiratory distress. Furthermore, there was a prespecified secondary outcome, namely, in-hospital mortality, number of patients sent to the ICU, number of patients requiring and duration of mechanical ventilation, and serum levels of 25-hydroxyvitamin D, total calcium, creatinine, and C-reactive protein (CRP). Patients were given a single oral dose of 20,000 IU vitamin D3 dissolved in a 10 ml peanut oil solution or a placebo of 10 ml. It was concluded that a single high dose was not more beneficial to hospitalized patients with COVID-19 than a placebo; hence, the use was not sufficiently supported [15].

In Mexico, Villasis-Keever et al. conducted a double-blind, randomized controlled trial involving 321 healthcare workers to assess the safety and efficacy of vitamin D supplementation in pandemic prevention from July 15 to December 30, 2020. Subjects were randomly receiving 4,000 IU vitamin D (VDG) or placebo (PG) for 30 days. Subsequently, participants were subjected to RT-PCR swab tests at baseline, and this was repeated when they experienced COVID-19 symptoms. Measurements of serum 25-hydroxyvitamin D3 levels and antibody tests were completed at baseline and on Day 45. The study found that the infection rate was lower in VDG than in PG (6.4% vs. 24.5%, P <0.001), as well as the risk of contracting the pandemic. In conclusion, vitamin D supplementation did prevent COVID-19 infection in exposed individuals [16].

The randomized, controlled, phase-3, open-label CORONAVIT trial involved 6,200 subjects without a history of vitamin D consumption at baseline. Subjects in the intervention group were offered a finger prick test to determine blood 25(OH)D concentration and provided with a six-month supply of low (800 IU daily, n=1,550) or higher dose (3200 IU daily, n=1,550) vitamin D with blood concentration of <75 mmol/L. The comparison group (n=3,100) did not receive blood vitamin D testing and supplementation. The groups were followed up in six months, and the primary outcome was intending to treat a proportion of subjects with at least one positive swab test or one doctor-confirmed respiratory tract infection. Among people 16 years old and older with a high baseline prevalence of suboptimal vitamin D concentration, the supplementation was not associated with risk reduction in all acute respiratory tract infections and COVID-19 [17].

Zinc

Zinc, an essential mineral found in cells throughout the body, aids in cell growth and division and is necessary for support for enzymes, proteins, and DNA. The human body cannot synthesize zinc and has to acquire the mineral from food and then stores it mostly in muscles and bones. Zinc plays a crucial role in the regulation of carbohydrate and lipid metabolism in addition to its involvement in signaling pathways that modulate the inflammatory response. Its significance in supporting the immune system stems from its indispensable contribution to leucocyte and lymphocyte proliferation, differentiation, maturation, and overall functioning. Additionally, zinc serves as an essential cofactor in various biological responses.

In Florida and Ohio, Thomas et al. reported a clinical randomized trial in which 214 RT-PCR-confirmed COVID-19 adults were recruited to determine the effects of high-dose zinc and ascorbic acid on the severity or duration of symptoms when compared to standard care. Subjects were randomized into four groups in a 1:1:1:1 allocation ratio to be given zinc gluconate 50 mg and ascorbic acid 8000 mg, both agents or standard care. The trial showed that patients without supplementation of zinc and ascorbic acid achieved 50% symptom reduction at a mean of 6.7 days vs. 5.9 with zinc, 5.5 with ascorbic acid, and 5.5 days with both (P = 0.45) [18].

Patel et al. coordinated a pilot, phase IIa, double-blind, randomized controlled trial to investigate the safety and feasibility of high-dose intravenous zinc (HDIVZn) in hospitalized patients. During recruitment and treatment, zinc concentrations were measured, and from the 33 participants, 15 received HDIVZn and 18 placebo. The participants were observed for adverse events throughout the week for 94 HDIVZn doses. Even though there were no significant adverse events reported by either group, three participants had infusion site irritation. The mean serum zinc on Day 1 was 7.7±1.6 in the HDIVZn group vs. 6.9±1.1 micromol/l, which is consistent with zinc deficiency. On Day 6, HDIVZn raised serum zinc levels above the cutoff value for deficiency at 10.7 mmol/l. This study inferred that zinc deficiency was common in hospitalized COVID-19 patients, and this was managed with HDIVZn safely with minimal side effects [19].

Selenium

Selenium is an essential trace element that is required for life, owing to its presence in selenoproteins as the unique amino acid selenocysteine [20-21]. It is mostly stored in the muscle tissue, but the thyroid gland carries the highest concentration because selenoproteins are necessary to assist with thyroid function. Adults older than 19 years old are recommended to consume 55 micrograms of selenium daily. Meanwhile, pregnant women require 60 micrograms, and lactating mothers need 70 micrograms daily. Selenoproteins and enzymes have antioxidant properties to help break down peroxides that may damage tissues and DNA. Studies indicated that the deficiency was a risk factor for severe disease progression, poor recovery, and COVID-19-related death [22,23].

A systematic review was conducted by Fakhrolmobasheri et al. to evaluate studies on the association between selenium levels and COVID-19. From the 11 studies reviewed, 10 used measurements of selenium levels, one utilized urinary selenium levels, and three measured selenium and selenoprotein levels. The outcomes assessed were severity, mortality, and risk of developing the pandemic. Based on the analysis of nine studies, it was inferred that lower selenium levels were associated with worse outcomes. However, two studies found no correlation between COVID-19 and serum selenium levels, and one study reported higher levels of urinary selenium in patients with severe and fatal cases of the pandemic. In general, deficiencies in selenium were linked to poorer outcomes, and individuals infected exhibited lower selenium levels compared to their healthy counterparts [24,25].

Asprinol

A randomized, multicentric, controlled clinical trial performed by Kumar et al. involving 260 mild to moderate patients assessed the safety and efficacy of APMV2020 tablets in relieving symptoms, accelerating clinical recovery, and reducing levels of these inflammatory markers: CRP, lactate dehydrogenase (LDH), and ferritin. The participants were adults 18-69 years old and were confirmed COVID-positive by RT-PCR within 48 hours of randomization. Of 260 participants, 99 were assigned the treatment, while 93 belonged to the control group. The treatment was APMV2020 tablets A and B, receiving a tablet twice a day for 10 days. Tablet A contained 150 mg of aspirin and 5 mg of promethazine hydrochloride, and Tablet B contained 2000 IU of vitamin D3, 750 mg of vitamin C, 80 mg of niacinamide, 15 mg of zinc sulfate monohydrate, 100 mcg potassium iodide, and 82.5 mcg sodium selenite.

Clinical manifestations assessed from baseline to Day 10 were fatigue, shortness of breath, cough, muscle pain, headache, diarrhea, and anosmia. Referring to WHO’s ordinal scale of 2 to 0 on Day 5, the manifestations were significantly improved in the two groups (P<0.05). However, the group given the treatment has earlier symptom relief than the controls. The treatment group had a significant decrease in serum LDH and ferritin levels (P<0.05) regarding the changes in inflammatory markers compared to the group.

In conclusion, the administration of APMV2020 tablets in patients yielded superior advantages to standard care. Faster recovery was seen in participants from the treatment group, as demonstrated by significantly reduced levels of CRP, LDH, and ferritin. This study has provided preliminary evidence for further analysis of micronutrients, such as vitamins and minerals, as well as aspirin in the management of COVID-19 [26].

Discussion

COVID-19 is an infectious disease compromising the immune system, and to achieve optimized immunity, immunomodulators are a necessity. Immunomodulation may be completed by applying a well-balanced diet containing vitamins C and D, minerals, zinc, and selenium. Currently, there is limited evidence to prove the efficacy of immunomodulators in COVID-19 even though numerous studies have been conducted and included in this review.

Evidence addressing the use of aspirin in the management of the pandemic has demonstrated reduced risk and 28-day mortality rate. Patients receiving aspirin also had shorter hospitalization duration and a higher probability of surviving. Trials showed that vitamin C improved oxygenation, respiratory rates, and laboratory parameters concerning vitamin C use in the treatment of patients.

Since the onset of the COVID-19 pandemic, vitamin D has garnered significant attention with diverse outcomes. Specifically, two trials indicated that the use of vitamin D could potentially be effective as a preventive measure. Other trials suggested that hospitalized patients did not experience significant benefits from high-dose vitamin D. Instead, these patients showed more favorable outcomes when zinc and selenium were utilized [5-7].

Zinc, as another agent, has received considerable attention and extensive study since the onset of the pandemic. Previous studies consistently observed that the patients were predominantly deficient in zinc. Additionally, a randomized controlled trial provided further evidence by showing that the administration of zinc facilitated a quicker reduction of symptoms [18,19].

Selenium is a trace element essential for the body in countering inflammation. Studies regarding the effects of the element on COVID-19 are still limited. A systematic review concluded that selenium deficiency was more prevalent in patients who contracted COVID-19. In hospitalized patients, individuals with selenium deficiency had worse outcomes. Other studies found no correlation between COVID-19 and selenium levels. Several clinical trials are required to assess the effects of selenium on infections and inflammations, specifically COVID-19 [24,25].

In a clinical trial conducted by Kumar et al., an innovative approach known as APVM2020 was proposed as a potential precise and tailored therapy for alleviating symptoms associated with COVID-19. This therapeutic approach involves combining two tablets, one containing aspirin and promethazine, and the other comprising vitamin D3, C, and B3 supplemented with zinc and selenium. The approach provides targeted relief for the symptoms experienced by patients. This intervention has been validated to build immunity to recover from infection. Assessments at baseline and on Days 5 and 10 have implied significant improvement of symptoms and inflammatory marker levels in hospitalized patients. Therefore, APVM2020 is a good candidate to be included in the COVID-19 management protocol [26].

## Conclusions

An imbalance in nutrition may trigger inflammation and compromise the immune system. This phenomenon is commonly found in patients with severe COVID-19, particularly in older, malnourished, obese individuals with comorbidities. Studies suggested that several vitamins and minerals (vitamins C, D, selenium, and zinc) were crucial in the regulation of inflammatory response. COVID-19 management includes medication, supportive therapy, anti-platelets, and also micronutrients. Previous studies have assessed the effects of important micronutrients, such as vitamins C, D, zinc, selenium, and aspirin in preventing and treating COVID-19, with promising results. Asprinol is a supplement containing important micronutrients and aspirin. Therefore, it is an important drug for the treatment of COVID-19 patients.
